# Host-Directed Therapies: Modulating Inflammation to Treat Tuberculosis

**DOI:** 10.3389/fimmu.2021.660916

**Published:** 2021-04-19

**Authors:** Stefanie Krug, Sadiya Parveen, William R. Bishai

**Affiliations:** Department of Medicine, Division of Infectious Diseases, Johns Hopkins University School of Medicine, Baltimore, MD, United States

**Keywords:** tuberculosis, PARP inhibition (PARPi), MMPs (metalloproteinases), immunotherapy, diphtheria fusion protein toxin, MDSCs, host-directed therapies

## Abstract

Following infection with *Mycobacterium tuberculosis*, the causative agent of tuberculosis (TB), most human hosts are able to contain the infection and avoid progression to active TB disease through expression of a balanced, homeostatic immune response. Proinflammatory mechanisms aiming to kill, slow and sequester the pathogen are key to a successful host response. However, an excessive or inappropriate pro-inflammatory response may lead to granuloma enlargement and tissue damage, which may prolong the TB treatment duration and permanently diminish the lung function of TB survivors. The host also expresses certain anti-inflammatory mediators which may play either beneficial or detrimental roles depending on the timing of their deployment. The balance between the timing and expression levels of pro- and anti-inflammatory responses plays an important role in the fate of infection. Interestingly, *M. tuberculosis* appears to manipulate both sides of the human immune response to remodel the host environment for its own benefit. Consequently, therapies which modulate either end of this spectrum of immune responses at the appropriate time may have the potential to improve the treatment of TB or to reduce the formation of permanent lung damage after microbiological cure. Here, we highlight host-directed TB therapies targeting pro- or anti-inflammatory processes that have been evaluated in pre-clinical models. The repurposing of already available drugs known to modulate these responses may improve the future of TB therapy.

## Introduction

Tuberculosis (TB) is a devastating communicable disease caused by *Mycobacterium tuberculosis* (*M.tb*) that is responsible for approximately 10 million infections and 1.4 million human deaths every year (1). Global TB control is complicated by long treatment durations and emerging drug resistance (1). Interestingly, most people infected with *M.tb* develop lifelong latent TB without ever experiencing signs and symptoms of disease. Successful containment is the result of a multifaceted immune response that restricts bacterial expansion but may fail to completely eliminate the pathogen (2). When sterilization is not achieved, the host may nevertheless successfully contain the infection by forming granulomas. However, in individuals who progress to active TB, granulomatous containment breaks down, resulting in lesion expansion, necrosis and liquefaction accompanied by bacterial proliferation and lung damage (2). This granulomatous inflammation during active TB may permanently diminish lung function even after completion of TB therapy (3).

The host utilizes both anti- and pro-inflammatory mechanisms in an effort to contain the infection: during latent *M.tb* infection, the immune response is successfully balanced but during active disease, this homeostatic balance is lost and disease progression occurs. Anti-inflammatory responses, mediated by regulatory T cells (Tregs), myeloid-derived suppressor cells (MDSCs), M2-polarized macrophages and cytokines such as interleukin (IL)-10, are observed during active TB and may antagonize the bactericidal effects of the immune system (4). Despite the presence of these immuno-tolerizing cells, host pro-inflammatory responses during active TB are often inappropriately expressed at high levels, either spatially or temporally, resulting in lung damage. Consequently, host-directed therapies (HDTs) that modify these non-productive immunologic responses may offer potential benefit as adjunctive agents alongside antimicrobial TB therapy (5). In this mini-review, we highlight FDA-approved drugs as well as select agents in development that have immunomodulatory activity and are under study as HDTs for TB in pre-clinical models and/or human clinical trials.

## Improving TB Therapy by Modulating Pro-Inflammatory Responses

**Figure 1:**
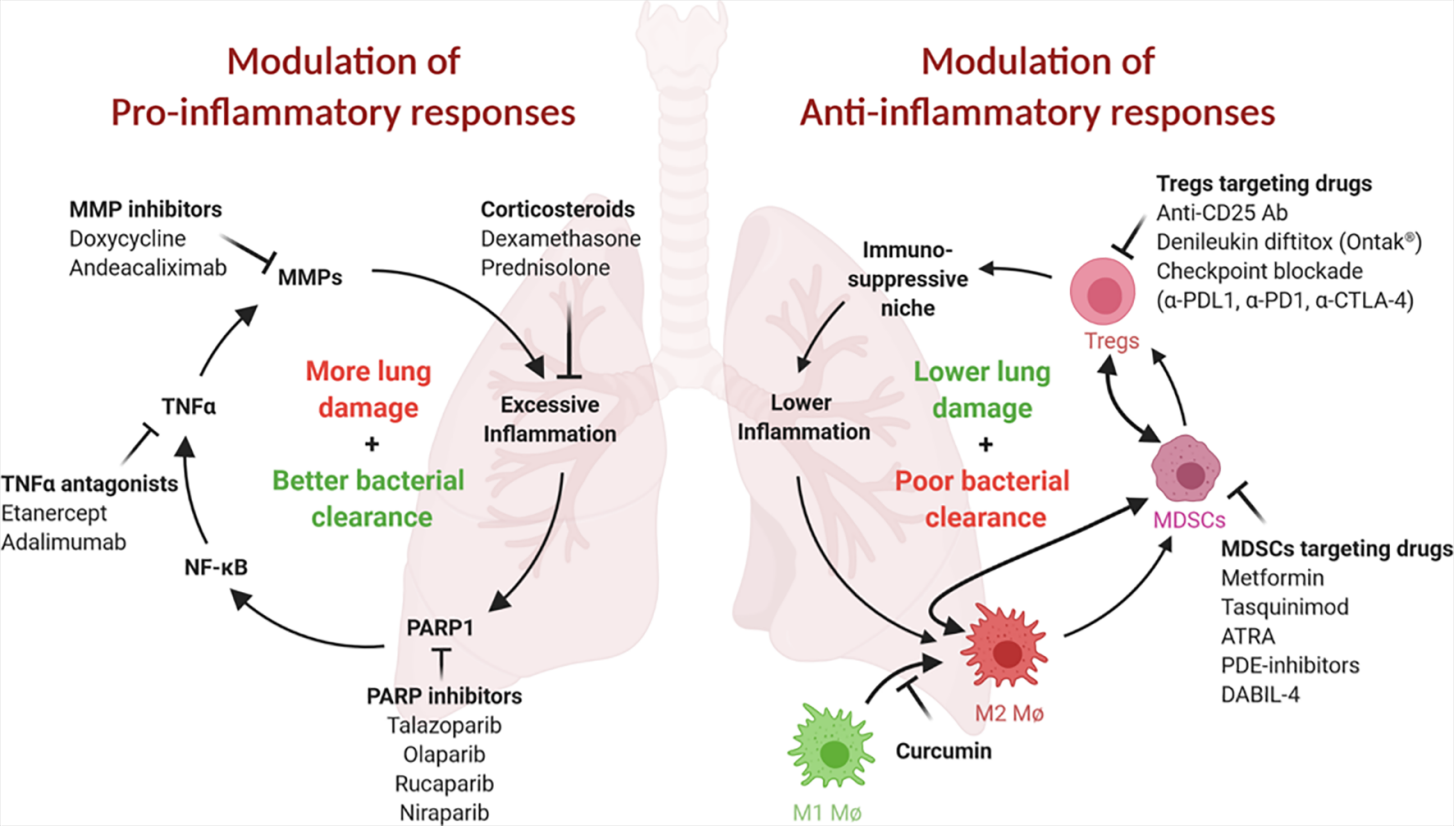
Both pro- and ani-inflammatory responses play critical roles in TB pathogenesis. (Left) Proinflammatory responses and tissue remodeling in TB are important for bacterial clearance but may lead to excessive inflammation and persisting lung damage. Adjunct modulation of lung remodeling (for example, *via* TNFα or MMP inhibition) or inflammation (for example, by corticosteroids) may improve the outcome of TB therapy. Inhibition of PARP1, an essential NF-κB, TNFα and MMP cofactor and driver of lung inflammation, may be similarly beneficial. (Right) Anti-inflammatory responses safeguard against tissue damage but may result in less than desirable bacterial clearance. These responses are often mediated by immunosuppressive cell populations, such as MDSCs, Tregs and M2 macrophages. Inhibition or elimination of these cell types may be achieved using the inhibitors shown. This figure was created using BioRender.

In immunocompetent patients with active TB, pro-inflammatory immune responses are often robust but fail to contain bacterial proliferation, leading to tissue damage and nonproductive inflammation. Nearly half of all active TB patients suffer from persistent or even progressive pulmonary dysfunction and face an increased risk of chronic lung disease even after microbiologically successful cure (3, 6–9). Post-TB lung defects (PTLD) include obstructive or restrictive lung disease, both of which may lead to chronic dyspnea, cough, reduced exercise tolerance, and a heightened risk for infections (3). In addition to shortening the duration of therapy, a parallel goal for TB HDTs is to avoid the development of irreversible lung damage from nonproductive inflammatory responses and to concomitantly improve the quality of life of TB survivors (3, 10). In this section, we discuss several classes of HDTs that may reduce nonproductive inflammation and PTLD (**Figure 1**, left; **Table 1**, top).

**Table 1:** Immune-modulatory drugs that may improve TB therapy.

Drug	HDT Class	Host Target	Applications	Preclinical data in TB	Ref.
Doxycycline	MMP Inhibitors	Multiple MMPs	Bacterial infections	Improved TB containment in cells, guinea pigs; Phase II trial ongoing (NCT02774993)	(11)
Marimastat	MMP Inhibitors	Multiple (MMP-1, -2, -7, -9, -14)	Cancer (discontinued)	Improved TB containment in mice	(12, 13)
Andecaliximab	MMP Inhibitors	MMP-9	Cancer, auto-inflammatory disorders (in development)	Reduced relapse rates in mice	(14, 15)
Cipemastat	MMP Inhibitors	MMP-1, -8, -13	Rheumatoid arthritis (discontinued)	Increased lung damage and death in mice; no effect in rabbits	(16, 17)
Etanercept	TNF antagonists	TNFα	Arthritis (various forms), ankylosing spondylitis	Accelerated bacterial clearance, reduced relapse rates in mice; may improve outcome in TB-HIV patients (Phase I) or severely ill TB patients; risk of impaired bacterial containment without adequate anti-TB therapy	(15, 18–22)
Dexamethasone/Prednisolone	Corticosteroids	Broad-spectrum anti-inflammatory effects *via* modulation of glucocorticoid/mineralocorticoid receptor signaling	Inflammatory and immune-mediated disorders (numerous)	Modest improvements in lung function; recommended for TB meningitis (survival benefit) but not for pulmonary TB	(23–31)
Talazoparib	PARP inhibitors	PARP1/2; PARP3, PARP4, TNKS1, TNKS2	Cancer	May reduce inflammation and TB lung damage in mice	(32–36)
Olaparib	PARP inhibitors	PARP1/2; PARP3, PARP4, PARP16, TNKS1, TNKS2	Cancer	N/A	(33, 34, 36)
Rucaparib	PARP inhibitors	PARP1/2, PARP3, PARP10, TNKS1, TNKS2	Cancer	N/A	(33, 34, 36)
Niraparib	PARP inhibitors	PARP1/2, PARP3, PARP4, PARP12	Cancer	N/A	(33, 34, 36)
Metformin	MDSCs	HIF1α, CD39, CD73, AMPK-DACHi-CXCL1	Diabetes	Reduced severity and mortality in diabetic patients	(37, 38)
Tasquinamod	MDSCs	S100A9	Cancer	Decreased lung and spleen bacillary burden in mice	(39)
ATRA	MDSCs	Upregulates glutathione synthase	Cancer	Decreased lung bacillary burden and pathology in mice and rats	(40–42)
DABIL-4	MDSCs	IL-4R	Preclinical model of breast cancer	Decreased lung bacillary burden in mice	(43)
Sildenafil	MDSCs	PDE-5i	Erectile dysfunction and pulmonary hypertension	Reduced lung bacillary burden, pathology and severity in mice	(44)
Roflumilast and CC-11052	MDSCs	PDE-4i	COPD	Improved lung function in mice	(45, 46)
Denileukin Diftitox (Ontak^®^)	Tregs	IL-2R	Refractory cutaneous T-cell lymphoma	Reduced lung bacillary burden in mice	(47)
Checkpoint blockade therapy	Tregs	CTLA4, PD1	Cancer	*Mtb*-infected macaques overexpress CTLA-4	(48)
Curcumin	M2 macrophages	IL-10	Preclinical models of cancer	Modest efficacy in mice	(49)
Anti-IL-10 antibody	Tregs	IL-10	Preclinical model of cancer	Reduced lung bacillary burden in mice	(50)

MMP, matrix metalloproteinases; TNKS, tankyrase; PDE, phosphodiesterase.

### MMP Inhibitors

Tissue-degrading matrix metalloproteinases (MMPs), in particular MMP-1, -3 and -9, are major drivers of TB-associated lung damage (51–55). While extracellular matrix remodeling is important for immune cell migration and granuloma formation, MMP levels in TB patients remain elevated even after treatment completion and thus may drive progressive lung dysfunction (55, 56). Consequently, adjunctive MMP inhibition has been studied as an HDT to improve TB outcome. **Doxycycline**, a well-known antibacterial agent, also is known to have MMP inhibitory properties, making it the only currently FDA-approved MMP inhibitor. Doxycycline has been shown to not only inhibit TB-induced MMP activation but also to contain mycobacterial growth in cells and guinea pigs (11). Results from a phase II pilot study (NCT02774993) that evaluated the efficacy of adjuvant doxycycline as a novel HDT for pulmonary TB are pending and may offer insights into the safety and efficacy of this approach.

Excess MMP activity is observed in a number of human degenerative diseases and hence several targeted MMP inhibitors have been developed and evaluated in human studies. While adverse effects hindered early MMP inhibitors, there is newfound optimism that this may be overcome with a newer generation of inhibitors (57). For example, the broad-spectrum MMP inhibitor **marimastat** (BB-2516) reduced granuloma formation and bacterial growth *in vitro* and increased the efficacy of TB antibiotics in mice but its clinical development was discontinued due to its side effects (12, 13). However, the humanized monoclonal MMP-9 antibody **andecaliximab** is in late-stage development for cancer and auto-inflammatory disorders (14) and might improve TB outcome since the addition of an anti-MMP-9 antibody has been shown to reduce TB relapse rates in mice (15). In contrast, the MMP-1 inhibitor **cipemastat** increased immunopathology and death in *M.tb*-infected C3HeB/FeJ mice and failed to prevent *M.tb*-mediated cavity-generation in a rabbit model (16, 17). Nonetheless, the next generation of MMP inhibitors with improved selectivity, specificity and safety is a promising class of drugs that warrants consideration for HDT activity in TB.

### TNF Antagonists

An alternative to direct MMP inhibition is to modulate the factors that promote MMP expression and TB inflammation, such as tumor necrosis factor α (TNFα) and the transcription factor NF-κB (55). TNFα is an important driver of TB lung damage by enhancing granuloma progression, cavitation, and MMP expression, and its expression levels are inversely correlated with the resolution of lung lesions during TB therapy (58–61). Correspondingly, HIV-positive TB patients generally have less lung damage than HIV-negative TB patients, and TB-immune reconstitution inflammatory syndrome (TB-IRIS) following antiretroviral therapy is associated with increased lung damage and reduced lung function (8, 62–64). TNFα also contributes to restrictive and obstructive airflow deficits by promoting fibrogenesis (18, 65, 66). Adjuvant administration of the TNFα antagonist **etanercept** accelerated bacterial clearance and reduced relapse rates in mice, and a promising phase I trial showed that etanercept may improve lung involvement and treatment responses in TB-HIV patients (15, 19, 20). There have also been case reports of TNFα inhibitors being used successfully to improve the clinical course of patients with advanced drug-susceptible TB who were doing poorly (21). In contrast, however, TNFα inhibitors are well-known to impair bacterial containment when used without accompanying multidrug anti-TB therapy (18, 22). While TNFα antagonists have the potential to improve TB therapy when used as adjunctive agents, there have been concerns about their expense, their need to be given parenterally, and the potential for disease worsening if administered without adequate anti-TB chemotherapy, and due to these concerns advanced clinical trials to test them as adjunctive HDTs for TB have not been performed (18, 60, 67).

### Corticosteroids

Corticosteroids are another class of anti-inflammatory drugs that have garnered attention as potential TB-HDTs (68). In pulmonary TB, adjunctive corticosteroids, including the broadly immunosuppressive agents **dexamethasone** and **prednisolone**, have been studied for their ability to reduce post-treatment morbidity. Indeed, while some studies have demonstrated modest improvements in clinical outcomes, such as preservation of lung vital capacity, major improvements in the prevention of lung disability have not been shown (23–28). Thus, corticosteroids are not recommended in current TB treatment guidelines for the management of pulmonary TB (29, 30). Corticosteroids have also been evaluated in the management of tuberculous pericarditis, but they do not appear to change outcomes and are currently not recommended in that setting (69). In contrast, well-controlled studies have demonstrated a clear-cut survival benefit for use of corticosteroids in TB meningitis, and hence corticosteroids are considered mandatory in the treatment of that form of TB (31).

### PARP Inhibitors

Poly(ADP-ribose) Polymerase (PARP) inhibitors (PARP-*I*s) are a new class of anticancer drugs introduced in the last decade, and four such agents are already FDA-approved. The PARP family of enzymes, comprised of at least 17 members, regulates wide-ranging cellular functions *via* the post-translational modification of mono- or poly(ADP-ribosyl)ation (70–74). PARP1, the founding member of the PARP family, is a eukaryotic master regulator particularly important for inflammatory processes and stress responses and accounts for at least 85% of cellular poly-ADP-ribose (PAR) formation (75). Importantly, PARP1 amplifies and sustains chronic inflammation by inducing inflammatory mediators that further stimulate its own activation (75, 76). Consequently, PARP1 contributes to disorders such as endotoxic shock, sepsis, asthma, COPD and ARDS, and PARP-*I*s have been shown to reduce inflammation and disease severity in numerous inflammatory conditions (75–78). PARP1 is an essential NF-κB, TNFα and MMP cofactor, and PARP-*I*s protect against tissue degradation by inhibiting multiple MMPs (71, 79–86). Therefore, PARP-*I*s have been proposed as HDTs for reducing TB-induced inflammation and lung disease (32). There are currently four FDA-approved PARP-*I*s for cancer therapies, **talazoparib** (Talzenna, Pfizer), **olaparib** (Lynparza, AstraZeneca), **rucaparib** (Rubraca, Clovis Oncology) and **niraparib** (Zejula, GlaxoSmithKline), with many more in various phases of development looking to expand their application in cancer therapy and beyond (33–35). Since it has been shown that PARP1 inhibition can ameliorate numerous inflammatory conditions, including rheumatoid arthritis, asthma, atherosclerosis and allergy-, toxicity- and injury-induced inflammation, the addition of a PARP inhibitor might similarly improve TB therapy by reducing inflammation and lung damage (75, 79, 87).

## Improving TB Therapy by Modulating Anti-Inflammatory Responses

An important theme in TB pathogenesis research in recent decades has been the observation that *M.tb* carries virulence traits that subvert normal host immune responses and lead to pathogen survival and/or proliferation. One such mechanism is the recruitment of immunosuppressive or tolerizing cells to the site of infection, resulting in blunted bactericidal responses and the expression of elevated levels of IL-10 which further promotes anti-inflammatory responses (88). Indeed, it has recently been shown that the microbial polypeptide ESAT6 is one mediator that promotes the differentiation of M1 macrophages into anti-inflammatory M2 macrophages (89). Other tolerizing, immunosuppressive cells that are recruited to the site of infection include MDSCs, Tregs and M2-polarized macrophages. In this section, we highlight the major cell types involved in these anti-inflammatory responses and discuss drugs that target them and may be candidate TB HDTs (**Figure 1**, right; **Table 1**, bottom).

### Myeloid-Derived Suppressor Cells (MDSCs)

**MDSCs** represent an immunosuppressive cell population increasingly recognized as an important driver of TB pathogenesis. MDSCs are comprised of two distinct subsets: polymorphonuclear MDSCs (PMN-MDSCs) and mononuclear MDSCs (M-MDSCs). In mice, PMN-MDSCs are defined as CD11b^+^ Ly6G^+^ Ly6C^low^ and M-MDSCs as CD11b^+^ Ly6G^-^ Ly6C^High^. In humans, MDSCs are identified as CD11b^+^ CD33^+^ HLA-DR^low∕neg^ cells (90, 91), and these are further subdivided into PMN-MDSCs by the markers CD14^−^ CD66b^+^ CD15^+^, and M-MDSCs as CD14^+^ (92–95). While the role of MDSCs in suppressing inflammation has been extensively studied in cancer, it is becoming increasingly evident that MDSCs play an important role in the establishment of chronic infections including TB. Clinical studies have revealed that levels of MDSCs are high in the blood and sputum of active TB patients at the time of diagnosis and that they decline in response to successful chemotherapy (96–98). This association suggests that MDSCs may play an important role in the pathogenesis of active TB pathology and its dysfunctional inflammatory processes. Further evidence comes from murine studies where the relative abundance of MDSCs has also been found to correlate with the TB susceptibility of a given mouse strain. Relatively high levels of MDSCs are observed in susceptible mouse strains, such as 129S2 and C3HeB/FeJ, while lower MDSC levels are found in relatively resistant strains, such as BALB/c and C57BL/6 (99). Multiple HDTs have been tested in both pre-clinical and clinical settings that (1) inhibit the recruitment, expansion or function of MDSCs; or (2) specifically or non-specifically deplete their population.

**Metformin**. The widely used diabetes drug **metformin** inhibits the frequency and recruitment of MDSCs in cancer by modulating the expression and activity of HIF-1α, CD39, and CD73 and the AMPK-DACH1-CXCL1 axis (100, 101). A widely cited study in 2014 revealed that metformin reduced disease severity and inflammation in mice and was retrospectively associated with a lower degree of disease severity in diabetic patients with active TB who happened to be taking metformin during TB treatment (37). Another retrospective study showed that metformin therapy reduces the elevated TB mortality observed in diabetics (38). In spite of these observations, long-term chemotherapy studies in mice have failed to demonstrate a significant beneficial effect of adjunctive metformin together with standard TB chemotherapy (102). Clearly, prospective human studies are needed, and the NIH has recently funded a prospective Phase 2A study of metformin in patients with TB (103).

**Tasquinimod** is an experimental quinoline-3-carboxamide drug that has been studied in human prostate cancer (104). It has been shown to slow tumor growth in murine cancer models and to reduce MDSC tumor infiltration (105). It is believed to act by binding to and inhibiting the activity of the S100A9 protein; S100A9 together with S100A8 are known to modulate myeloid cell activity though TLR4 binding (104, 106). Because of its anti-MDSC properties, tasquinimod has been tested in murine TB models, and it has been shown not only to deplete MDSCs but also to decrease the relative bacterial burden in both lungs and spleens of infected animals (39).

**All-trans Retinoic Acid (ATRA, tretinoin, a vitamin A derivative)** is an FDA-approved drug which has been tested extensively in cancer models and has been shown to deplete MDSCs and slow tumor growth. While its precise mechanism of action is unknown, ATRA upregulates glutathione synthase (GSS), neutralizes high levels of reactive oxygen species (ROS) and induces differentiation of myeloid cells away from the MDSC phenotype (107). Importantly, however, ATRA has pleotropic effects on numerous cell types so in instances where it was found to be effective, one cannot be certain that its efficacy was through MDSC inhibition. Multiple groups have tested the effects of ATRA in murine TB models both as a monotherapy and in combination with standard TB therapy. In *M. tb*.-infected mice and rats, ATRA has been shown to reduce relative bacterial burden and lung pathology in a manner that correlates with MDSC depletion. The drug also exhibits anti-mycobacterial activity *in vitro* (96, 108).

In addition to the non-specific depletion of MDSCs, our group has recently tested the diphtheria toxin-related IL-4 fusion protein, **DABIL-4**, as a targeting agent against MDSCs which are known to express the IL-4 receptor, CD124. In an acute murine model of TB, DABIL-4 administration depleted IL-4R^+^ MDSCs, IL-4R^+^ M2 macrophages and IL-4R^+^ lymphocytes. Depletion of these cell populations coincided with a significant reduction in the lung bacillary burden at day 21 post infection (43). We have also tested DABIL-4 in a murine breast cancer model and demonstrated that targeted depletion of MDSCs results in slower tumor growth and reduced splenomegaly and metastasis (109).

**Phosphodiesterase inhibitors. Sildenafil**, an FDA-approved type 5 phosphodiesterase-selective inhibitor (PDE-5i), is used in human patients for the treatment of erectile dysfunction and pulmonary hypertension. The drug downregulates arginase-1 and nitric oxide synthase-2 (NOS2) in a cGMP-dependent fashion, thereby hampering the immunosuppressive potential of MDSCs (110). Maiga et al. showed that the combination of sildenafil and **cilostazol** (an FDA-approved PDE-3 inhibitor) reduced pathology, disease severity and bacterial burden in murine TB; however, monotherapy with sildenafil alone showed no statistically significant benefit in the same mouse model (44, 111). PDE-4 inhibitors, such as **roflumilast** and **CC-11052**, a Celgene PDE4 inhibitor in development, have also shown promising activity against TB in animal models (45, 46). A clinical trial evaluating CC-11052 as an adjunctive HDT alongside standard therapy has been conducted (NCT02968927), and preliminary results suggest that use of CC-11052 was associated with improvements in lung function (112).

### Regulatory T-Cells (Tregs)

Tregs comprise an immunosuppressive CD4^+^ T-cell population which express CD25 and FoxP3. CD8^+^ Tregs also exist but their role in TB has not been extensively studied. Classic CD4^+^ CD25^+^ FoxP3^+^ Tregs are anti-inflammatory cells which keep effector T-cell function in check while promoting MDSC recruitment and maturation to further facilitate immunosuppression. Their presence in active TB is believed to inhibit anti-bacterial immune responses and to contribute to disease progression (113). Consistent with this, elevated Treg levels have been described in the blood and pleural fluid in pulmonary TB patients compared with healthy controls, and Treg levels were observed to decline to healthy control levels after successful TB chemotherapy (114).

**Treg-depleting immunotherapies**. The administration of **anti-CD25 monoclonal antibodies** in various cancer models has not only depleted Tregs but also slowed tumor progression (115). Anti-CD25 antibodies have been tested in the mouse TB model and were found to reduce relative bacillary loads in the lung and spleen and to improve lung pathology (116). **Denileukin diftitox (Ontak^®^),** a diphtheria toxin-related IL-2 fusion protein that was previously approved by the FDA for the treatment of refractory cutaneous T-cell lymphoma, is known to have potent Treg-depleting activity and has also been tested in murine TB models (47, 117). Ontak^®^ monotherapy not only decreased Treg and MDSC frequencies in lungs and spleens but also significantly reduced relative bacterial CFU counts in a short-term TB mouse model. Additionally, the fusion protein toxin when combined with standard TB therapy significantly accelerated bacterial clearance in mice (47, 117).

### Checkpoint Blockade Immunotherapy

**Checkpoint blockade therapies,** such as anti-PD-1 and anti-CTLA4 antibodies, have revolutionized the field of immunotherapy and have become an essential part of standard care for various human malignancies (118). In *M.tb*-infected macaques, Tregs have been shown to express CTLA-4, suggesting that anti-CTLA-4-directed checkpoint inhibitors may offer a potential HDT TB treatment (48). However, several groups have reported TB reactivation in cancer patients treated with checkpoint blockade therapy (119–121). While this does not necessarily indicate that checkpoint inhibitors given as adjuvants alongside appropriate anti-TB chemotherapy will fail to accelerate TB cure, more studies will be needed reach a conclusion regarding the efficacy of checkpoint blockade therapy as HDT for TB.

### Anti-IL-10 Therapies

IL-10 is a key anti-inflammatory cytokine secreted by CD4^+^ T cells, macrophages and MDSCs that suppresses T-cell function, blunts inflammatory responses, and promotes TB disease progression (50). IL-10 has been implicated in the M2-polarization of macrophages and this may further contribute to anti-inflammatory responses. An abundance of M2-polarized macrophages has been described in human lung granulomas (122), although it remains unclear if these M2 macrophages are causal in granuloma formation or rather a secondary consequence. IL-10 inhibitors would be expected to inhibit the direct anti-inflammatory effects of IL-10 and also prevent conversion of M1 macrophages into M2 macrophages. Indeed, IL-10 inhibitors have been tested both in cancer models and also in models of TB. **Curcumin** (diferuloylmethane), one of the active compounds found in turmeric, has been shown to modulate IL-10 levels and the frequency of M2 macrophages (123). Preparations of curcumin have been shown to drive a therapeutic benefit in a murine metastatic breast cancer model (124). In the context of TB, curcumin has also been shown to control the growth of *M.tb* in THP-1 macrophages and in primary alveolar macrophages derived from healthy human controls (125). More recently, a nanoparticle preparation of curcumin was tested in a murine TB model where it showed modest activity as monotherapy and more potent activity in combination with isoniazid (49). Direct inhibition of IL-10 with an **anti-IL-10-receptor antibody** in a murine TB model was shown to reduce bacterial CFU counts although it had little impact on the lung pathology (50).

## Discussion

Host-directed therapies have the potential to improve the treatment of TB by modulating either pro- or anti-inflammatory immune mechanisms. Interference with certain pro-inflammatory mechanisms offers the potential to reduce lung damage, increase antibiotic efficacy and shorten treatment duration. On the other hand, modulation of certain immunosuppressive immune responses may enhance the innate bactericidal activity of the immune system and thus accelerate bacterial clearance. Repurposing drugs that are safe and approved for human use is an approach that may fast-track the clinical development of new host-directed TB treatment regimens. Here, we reviewed HDTs of interest for TB that target pro- or anti-inflammatory immune mechanisms (**Figure 1**; **Table 1**). On the proinflammatory side, we highlighted MMP inhibition, TNFα antagonists, corticosteroids and PARP inhibition to reduce TB-associated lung damage and inflammation. However, immune modulation in TB should be approached with caution as disrupting the intricate host-pathogen relationship can also increase the risk for disease progression or exacerbate inflammation. It is important that the dosing, frequency and timing of TB-HDTs are carefully optimized to minimize potentially harmful effects. Moreover, HDTs should be primarily evaluated as treatment adjuvants to be utilized alongside fully active traditional anti-TB chemotherapy. A related concern is that of drug-drug interactions and the potential for one agent to reduce the circulating concentration of another.

Even though TB-associated persistent lung dysfunction is a common disability in TB survivors, there are currently no guidelines for the diagnosis or management of PTLDs, and it is unclear to what extent they contribute to the economic burden of TB (8, 126). Reducing TB-associated lung dysfunction has the potential to greatly improve the quality of life after TB by reducing morbidity and loss of income. While pulmonary function testing in early TB carries some risk of TB transmission, it has been safely implemented in numerous clinical trials. We therefore recommend that more consideration should be given to the routine assessment of lung function in TB clinical trials. In addition to HDTs, non-pharmacological interventions, such as pulmonary rehabilitation, may improve lung function after completion of TB therapy and should be considered in the management of TB patients on a case-by-case basis (127). Importantly, we hope to increase awareness that the fight against TB does not end with microbiological cure.

## Author Contributions

SK and SP contributed equally to this mini-review. All authors contributed to the article and approved the submitted version.

## Conflict of Interest

The authors declare that the research was conducted in the absence of any commercial or financial relationships that could be construed as a potential conflict of interest.
